# The role of YKL-40 in Alzheimer’s disease pathology and drug targeting

**DOI:** 10.7717/peerj.21361

**Published:** 2026-06-08

**Authors:** Yaru Sha, Hong Fu, Kun Lu, Guohui Wang, Yubing Wang

**Affiliations:** 1 School of Life Science and Technology, Shandong Second Medical University, Weifang, Shandong, China; 2 Translational Medical Center, Weifang No.2 People’s Hospital, Weifang, Shandong, China

**Keywords:** Alzheimer’s disease, YKL-40, CHI3L1, Neuroinflammation, Drug targets

## Abstract

**Background:**

Alzheimer’s disease (AD) is a progressive neurodegenerative disorder characterized by the accumulation of amyloid-beta (Aβ) plaques, hyperphosphorylated tau tangles, and significant neuronal loss. Recent studies have implicated YKL-40, a glycoprotein commonly associated with inflammation and neural apoptosis, in the pathogenesis of AD.

**Methods:**

We conducted extensive searches across major scientific databases, including PubMed, Web of Science, and Embase. We selected peer-reviewed articles, review articles, and clinical studies focusing on YKL-40 in AD.

**Results:**

This review comprehensively analyses the multifaceted role of YKL-40 in AD, covering its cellular localization, biomarker associations, and pathological mechanisms. We also summarize the mechanistic pathways by which YKL-40 contributes to disease progression, highlighting its role in neuroinflammation, neural apoptosis, and disruption of the circadian regulation of immune responses. Moreover, the development of drugs that target YKL-40, such as humanized anti-YKL-40 antibodies and small molecules, offers promising strategies for blocking AD progression.

**Conclusion:**

This review highlights the potential of YKL-40 as a novel drug target and its implications for enhancing diagnostic precision and treatment strategies in combating Alzheimer’s disease.

## Introduction

Alzheimer’s disease (AD), the foremost cause of dementia, is a highly debilitating and relentlessly progressive neurodegenerative disorder. Clinically, AD is characterized by a progressive decline in cognitive function, with memory loss being one of the earliest and most prominent symptoms (Cacace, Sleegers & Van Broeckhoven, 2016). The pathology of AD involves a complex web of interrelated processes. The formation of extracellular amyloid plaques, which are composed of amyloid-beta protein (Aβ), is a hallmark feature (Guo et al., 2022). The accumulation of Aβ disrupts the neuronal microenvironment and activates microglia and astrocytes, the immune cells of the brain. This activation aims to clear plaques but can lead to the release of inflammatory mediators that damage neurons (Thal et al., 2002). The abnormal phosphorylation of tau proteins within neurons results in the formation of neurofibrillary tangles (NFTs), which disrupt microtubules crucial for intracellular transport. This impairs axonal transport, depriving neurons of essential nutrients and molecules, resulting in dysfunction and death (Chacón, Irineo-Moreno & Loera-Valencia, 2025). The loss of NFTs and synapses, especially in the hippocampus and cerebral cortex, directly correlates with cognitive decline. The hippocampus, which is vital for memory, degenerates, and the resulting synapse loss disrupts neural circuitry and information processing (Donev et al., 2009; Leng & Edison, 2021).

Neuroinflammation is a key factor in most neurodegenerative diseases, including AD. Neuroinflammation can be triggered by nerve damage ((for example from trauma, ischemia, or toxins), infections, or autoimmune reactions. In AD, chronic neuroinflammation starts and persists (Forloni & Balducci, 2018). The International Working Group and the National Institute on Aging-Alzheimer’s Association used increased Aβ and elevated total tau (T-tau) and phosphorylated tau (p-tau) levels in cerebrospinal fluid (CSF) as diagnostic criteria. Additionally, molecules such as YKL-40, BACE, neurofilament, α-synuclein, and neurogranin are being researched as potential biomarkers (Jack et al., 2018; Klyucherev et al., 2022; Liu, Zhang & Wang, 2024). YKL-40, also known as Chitinase-3-like protein 1 or CHI3L1 is a member of the chitinase family, and is elevated in both protein and RNA levels in AD (Querol-Vilaseca et al., 2017). YKL-40 upregulation is also observed in other neurodegenerative diseases, such as encephalitis (Bonneh-Barkay et al., 2008), multiple sclerosis (Hinsinger et al., 2015), glioblastoma (Qin et al., 2017), traumatic brain injury (Wiley et al., 2015) and Parkinson’s disease (Xie et al., 2021), and is correlated with markers of neuronal injury and synaptic destruction. Investigations comparing inflammatory markers in CSF and peripheral blood across AD, mild cognitive impairment (MCI), and control groups revealed significantly elevated levels of YKL-40 and other inflammatory mediators (IL-6, sTNFR1, sTNFR2, IL-1β, MCP-1, sTREM2, *etc.*) in AD and MCI patients (Choi, Lee & Suk, 2011; Wang et al., 2023; Scheltens et al., 2021; Su, Bai & Zhang, 2016). Despite growing interest in the role of YKL-40 in AD, the literature remains fragmented, while some studies have focused on its utility as a biomarker; others have explored its mechanistic involvement in neuroinflammation, Aβ deposition, or blood−brain barrier compromise, but few have synthesized these findings into a cohesive framework. Additionally, its potential as a therapeutic target remains underexplored in integrative reviews.

In addition to being a mere biomarker for AD diagnosis, YKL-40 serves as a signalling molecule integral to the activation of astrocytes and microglia, which are pivotal in mediating the intricate processes of neuroinflammation. This review explores the biological functions of YKL-40, its role in the pathogenic cascade of AD, and its potential as a therapeutic target and prognostic indicator. The roles of YKL-40 are multifaceted: it is involved in the deposition of Aβ plaques, contributes to the compromise of the blood−brain barrier, and is associated with neuronal damage and loss. The involvement of YKL-40 in these key AD-related phenomena underscores its critical roles in disease onset and progression. Additionally, its presence and levels may have prognostic implications for AD, potentially aiding in assessing disease advancement and outcomes.

## Search Methodology

To ensure comprehensive, unbiased, and systematic coverage of the literature on YKL-40 in AD, this review followed a structured search and selection protocol. A multi-database search was conducted to capture a broad spectrum of relevant scientific literature, including PubMed, Web of Science and Embase, and articles were selected based on their coverage of medical, biological, and clinical research. The search was not restricted by a specific publication timeframe to include both foundational and latest research, although priority was given to peer-reviewed articles to maintain the clarity and accessibility of the methodological and result details.

A combination of MeSH terms (for PubMed) and keyword strings (for Web of Science and Embase) was used to minimize missed relevant studies, with core search terms organized around two thematic clusters, YKL-40 and Alzheimer’s disease. YKL-40-related terms included “YKL-40”, “Chitinase-3-like protein 1”, “CHI3L1”, and “YKL40 protein”, whereas AD-related terms included “Alzheimer’s disease”, “AD”, “Alzheimer disease”, and “Senile dementia of Alzheimer type (SDAT)”. These clusters were combined *via* the Boolean operator “AND” to form the core search strategy (YKL-40 OR Chitinase-3-like protein 1 OR CHI3L1 OR YKL40 protein) AND (Alzheimer’s disease OR AD OR Alzheimer disease OR Senile dementia of Alzheimer type OR SDAT).

**Figure 1:**
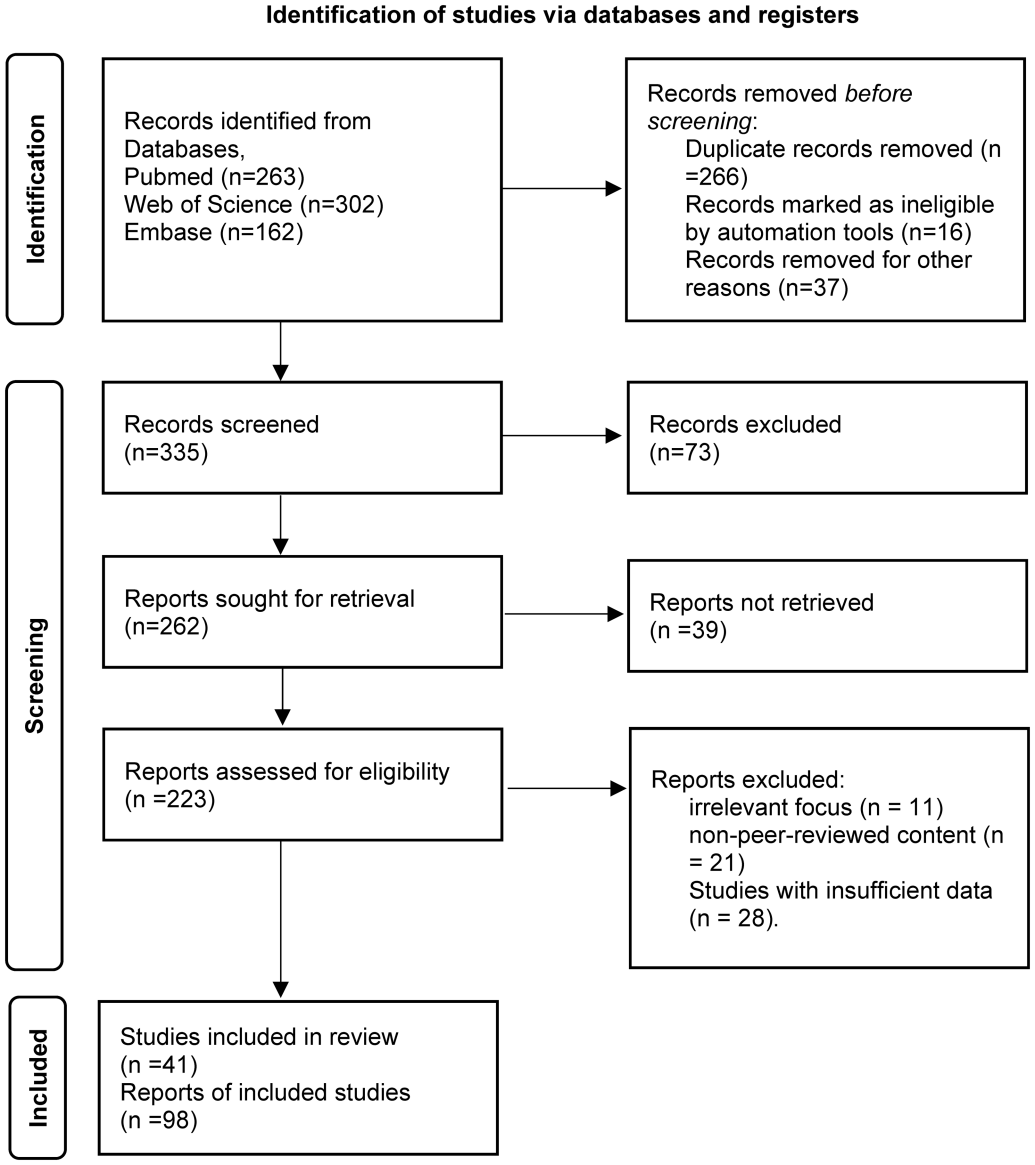
Flowchart for the initial database search and the final selection of articles for inclusion in the review.

The retrieved articles were screened *via* predefined inclusion and exclusion criteria to ensure their relevance to the role of YKL-40 in AD pathology. The inclusion criteria included peer-reviewed studies (primary research, reviews, and clinical studies comparing YKL-40 levels in AD patients and controls) and an explicit focus on YKL-40 (or CHI3L1) in AD patients (covering expression, molecular interactions, or biomarker potential). The exclusion criteria included studies with an irrelevant focus (YKL-40 not central or AD not the target disease), non-peer-reviewed content (preprints, abstracts, *etc.*), duplicate studies, and those with insufficient data on the role of YKL-40 in AD (Fig. 1).

## Correlation of YKL-40 with AD pathology

### Expression and cellular localization of YKL-40 in the brains of AD patients

Chitinase-3-like protein 1 (CHI3L1, YKL-40), a molecule expressed and secreted by a plethora of cell types, including neutrophils, differentiated macrophages, chondrocytes, synovial cells, vascular endothelial cells, and notably, astrocytes, has garnered attention for its role in AD. Astrocytic expression of YKL-40 is a focal point in the brain’s pathological landscape (Bonneh-Barkay et al., 2012a). Compared with those in controls, elevated levels of YKL-40 were detected in the cerebrospinal fluid and peripheral blood of AD and MCI patients, highlighting its potential as a biomarker (Choi, Lee & Suk, 2011; Jany et al., 2015). Immunohistochemical analyses revealed its localization in reactive astrocytes proximal to amyloid plaques (Kim et al., 2024), whereas *in vivo* findings associated it with neuroinflammatory responses (Craig-Schapiro et al., 2010).

Astrocytes are the most abundant cell type in the mammalian central nervous system (CNS) and play a key role in central nervous system processes, including neuronal excitability, synaptic development, and plasticity. Astrocytes are critical in a variety of central nervous system diseases, including autoimmune inflammation and degenerative diseases such as MS and AD. Glial fibrillary acid protein (GFAP), a hallmark of mature and reactive astrocytes, exhibits substantial colocalization with YKL-40, particularly in the frontal cortex, irrespective of the pathological state; an 80% colocalization rate with YKL-40 reinforces the latter as a reactive astrocyte marker (Querol-Vilaseca et al., 2017). In contrast, no significant overlap with neuronal MAP2 or microglial IBA-1 markers was observed. The temporal dynamics of YKL-40 expression after cortical contusion injury (CCI) further corroborate its induction during acute neuroinflammatory phases, which aligns with the expression profiles of other inflammatory mediators (Bonneh-Barkay et al., 2010). The predominant astrocytic localization of YKL-40 and its reactive expression patterns in pathological states suggest that YKL-40 plays a role as a sentinel of reactive gliosis in the context of neuroinflammation.

Microglia, as integral components of the CNS innate immune response, represent approximately 10% of all CNS cellular constituents (Lawson et al., 1990). These cells serve as the CNS’s primary and most immediate line of defense, playing a crucial role in the instigation and progression of neuroinflammation. Specifically, in the early stages of AD, microglial accumulation is observed within neocortical-aged plaques (Rogers & Lue, 2001), with their activation peak coinciding with the onset of Aβ deposition. Intriguingly, animal model studies have revealed that the YKL-40 protein may modulate microglial cell density within the brain. Notably, YKL-40 deficiency has been linked to increased microglial activation, coupled with a diminished capacity for Aβ phagocytosis and clearance, leading to the aggregation of Aβ plaques (Lananna et al., 2020). These findings are mirrored in clinical observations where elevated YKL-40 levels in the CSF of AD patients are associated with impaired microglial phagocytic function, thereby contributing to Aβ accumulation. Additionally, YKL-40 knockout (KO) mice presented a slight increase in IBA1 staining, indicating that YKL-40 may exert a regulatory influence on microglial activation (Lananna et al., 2020).

In addition to glial cells, neurons, the foundational units of nervous system architecture and functionality also exhibit associations with YKL-40. Through immunohistochemical approaches, the presence of YKL-40 has been identified in rare neuronal populations within the white matter (Craig-Schapiro et al., 2010). In another study, the binding of YKL-40 to CRTH2 inhibited the proliferation of neural stem cells (NSCs) and the differentiation of neurons, thereby suppressing hippocampal neurogenesis (Yang et al., 2025). Furthermore, by examining clinical spectra across a cohort of 121 individuals *via* PET imaging to measure tau and YKL-40, researchers reported a positive correlation between elevated CSF YKL-40 levels and increased tau-PET burden (Ferrari-Souza et al., 2022). This evidence suggests that YKL-40 may influence neuronal function during AD progression and potentially act as an accelerant in disease pathogenesis.

Therefore, the collective body of research underscores the multifaceted roles that YKL-40 plays within the CNS, particularly in AD. From the modulation of astrocytic and microglial activation to potential implications for neuronal structural integrity, YKL-40 has emerged as a protein of significant interest that may contribute to the intricate molecular interplay of AD pathology.

### Interplay of YKL-40 with core AD pathological markers

#### YKL-40 and the disequilibrium of Aβ production and clearance

The hallmark neuropathological manifestations of AD are the prolific buildup and deposition of Aβ peptides, accompanied by increased expression of proinflammatory mediators in the vicinity of both Aβ plaques and neurofibrillary tangles (NFTs). It is widely accepted that the aggregation of Aβ is a precursor to the neuroinflammatory cascade in AD, which, in conjunction with hyperactivated astrocytes and microglia, exacerbates the entrenchment of Aβ and tau proteins (Di Benedetto et al., 2022; Kwon & Koh, 2020; Joly-Amado et al., 2020). This deleterious cycle promotes the recruitment of immune cells and exacerbates neurotoxicity, which is correlated with cerebral atrophy and the deterioration of cognitive faculties (Di Benedetto et al., 2022; Burgaletto et al., 2020).

The presence of YKL-40-positive cells is a common observation around the periphery of Aβ plaques and within the vascular structures associated with Aβ deposition (Bonneh-Barkay et al., 2010; Llorens et al., 2017a). Immunohistochemical studies of AD-afflicted cerebral tissue revealed clusters of activated microglia and reactive astrocytes in close proximity to Aβ aggregates (Llorens et al., 2017b). When YKL-40 was deleted, amyloid plaque deposition was reduced in AD model mice (Lananna et al., 2020). Notably, the abrogation of YKL-40 expression in murine models of AD has been associated with a reduction in amyloid plaque burden, suggesting that the absence of YKL-40 tempers astrocytic activation and potentially enhances the clearance of Aβ plaques by microglia. An examination of the interplay between Aβ biomarkers and cognitive health revealed a positive correlation between YKL-40 and Aβ40 levels (Preis et al., 2024). Furthermore, quantification of YKL-40 in the CSF and serum of AD patients revealed a significant increase, with YKL-40 expression correlating positively with the hallmark Aβ protein of AD, inversely with cortical thickness, and displaying a direct relationship with the cognitive decline of AD patients (Wilczyńska et al., 2021). Thus, mitigating YKL-40 expression may offer a therapeutic avenue for reducing Aβ production and decelerating AD progression.

#### Interaction of YKL-40 with hyperphosphorylated tau in inflammatory pathogenesis

Biomarkers such as CSF total tau and phosphorylated tau have shown robust utility in tracking neuronal damage and disease progression (Dubois et al., 2014). Clinically, elevated levels of CSF t-tau are indicative of AD and span the spectrum of disease states, from asymptomatic to dementia (Bloudek et al., 2011; Ferreira et al., 2014). Similarly, CSF p-tau levels correlate with tau pathology and the presence of neurofibrillary tangles (Iqbal et al., 1986). In neurodegenerative diseases such as AD, YKL-40 is expressed in microglia and astrocytes close to Aβ plaques and is positively correlated with t-tau and p-tau, indicating the key role YKL-40 plays in AD (Craig-Schapiro et al., 2010; Querol-Vilaseca et al., 2017). Moreover, YKL-40 correlates positively with t-tau and p-tau at the preclinical stage of AD, and elevations in CSF YKL-40 and t-tau and p-tau proteins, particularly in Aβ-positive individuals, have been associated with the intensification of neuroinflammation (Nordengen et al., 2019). Elevated CSF YKL-40, concomitant with t-tau and p-tau, denotes a more pronounced inflammatory state in Aβ-positive patients across the clinical continuum of AD, suggesting its potential complexity in exacerbating neuroinflammation (McGrowder et al., 2021). Furthermore, the alignment of YKL-40 levels with tau pathology highlights the importance of elucidating the underlying mechanisms of this association (Querol-Vilaseca et al., 2017).

Accumulating evidence has confirmed that YKL-40 indirectly regulates tau hyperphosphorylation and aggregation primarily by mediating neuroinflammation and inducing neuronal dysfunction, with the underlying mechanisms falling into two core aspects (Andronie-Cioara et al., 2023; Mwale et al., 2025; Trudel et al., 2025; Wang et al., 2015; Wei et al., 2017; Zeng et al., 2023). First, YKL-40 activates microglia and astrocytes to trigger sustained neuroinflammation, which in turn modulates tau pathology. YKL-40 binds to the CD44 receptor on microglia and activates the NF-κB pathway, upregulating the secretion of pro-inflammatory cytokines such as IL-1β and TNF-α (Trudel et al., 2025). These factors subsequently activate tau kinases including GSK-3β and CDK5, and inhibit the activity of PP2A, a key tau phosphatase, resulting in imbalanced tau phosphorylation and tau accumulation (Wang et al., 2015). Meanwhile, YKL-40 activates the ERK/p38 MAPK pathway in astrocytes, promoting the release of ROS, NO, and chemokines (Mwale et al., 2025). This induces neuronal oxidative stress and enhances tau aggregation, forming an inflammatory amplification loop. Activated astrocytes further suppress PP2A activity and disrupt the blood–brain barrier, amplifying the regulatory effects of inflammation on tau pathology (Trudel et al., 2025).

Second, YKL-40 induces neuronal dysfunction and disrupts tau metabolic homeostasis. YKL-40 mediates excitotoxicity through neuroinflammation and downregulates the expression of synaptophysin and PSD-95, leading to synaptic degeneration and impaired intracellular tau transport and accumulation (Zeng et al., 2023). Furthermore, YKL-40 activates the AKT/mTOR pathway to inhibit autophagic flux, reducing the degradation of hyperphosphorylated tau and facilitating its aggregation into neurofibrillary tangles (NFTs) (Mwale et al., 2025). It also activates neuronal calcium channels to trigger calcium homeostasis imbalance, which promotes abnormal tau phosphorylation *via* calpain (Wei et al., 2017). The neuroinflammation and neuronal dysfunction mediated by YKL-40 act synergistically to form a vicious cycle of “neuroinflammation–neuronal injury–tau pathology” (Trudel et al., 2025). DAMPs such as ATP and HMGB1 released from damaged neurons, as well as tau aggregates themselves, can further activate neuroinflammation and accelerate the progression of AD (Andronie-Cioara et al., 2023).

In summary, YKL-40 promotes gliosis and neuroinflammation, impairs neuronal function, and collaboratively causes imbalanced tau phosphorylation and aggregation, thereby driving the pathological progression of AD (Andronie-Cioara et al., 2023; Mwale et al., 2025; Trudel et al., 2025; Wang et al., 2015; Wei et al., 2017; Zeng et al., 2023). Elucidating the mechanisms underlying their association will provide novel insights for the early diagnosis and targeted therapy of AD, and exploring their interactive targets remains an important direction for future AD research.

#### Synergistic neurodegenerative effects of YKL-40 and α-Syn

The presynaptic neuronal protein α-synuclein (α-Syn), recognized for its pivotal contribution to neurodegenerative pathology, constitutes the primary building block of neuronal inclusion bodies and serves as a cardinal marker of neuroinflammation (Tu et al., 2021; Panicker et al., 2021; Cui et al., 2025). The accumulation of α-Syn within neurons is a hallmark of neuroinflammatory pathology. AD pathogenesis has been linked to the aggregation of impaired mitochondria within neuronal cells (Hashimoto et al., 2003). Cellular senescence is coupled with diminished lysosomal autophagy, resulting in the accumulation of Aβ and α-Syn oligomers on mitochondrial membranes, subsequently instigating cytochrome C release and activating a caspase cascade, ultimately leading to pronounced neurodegeneration. A positive correlation between CSF levels of α-Syn and the inflammatory marker YKL-40 has been established, with elevated levels of both proteins associated with increased markers of neuronal damage within the CSF (Hall et al., 2018). α-Syn has been shown to act in concert with tau (Giasson et al., 2003) and Aβ (Marsh & Blurton-Jones, 2012), facilitating their accumulation, suggesting that α-Syn may trigger neuroinflammatory responses, exacerbating neurodegeneration.

#### YKL-40 and GFAP reflect distinct astrocyte subpopulations and their unique spatial distributions

GFAP serves as the quintessential intermediate filament within mature astrocytes and is vital for the structural integrity of the cytoskeletal network during astrocyte maturation. The modulation of GFAP expression, accompanied by its cytoarchitectural reorientation, plays a critical role in the pathophysiology of a multitude of CNS disorders, ranging from inflammatory to neurodegenerative processes (Li et al., 2020). Investigations into the early stages of AD have revealed disease-specific astrocyte populations in murine models, a finding echoed by the observation of increased astrocytic proliferation in postmortem human AD brains. Transcriptomic scrutiny of cortical samples from AD patients has revealed a prominent increase in YKL-40 within astrocytes, albeit with a minor increase in microglia (Lananna et al., 2020). Cells exhibiting YKL-40 positivity constitute a unique subset within the astrocytic cadre. Experimental evidence underscores a surge in YKL-40 expression aligned with astrocytic maturation in the developing rodent brain, suggesting that YKL-40 overabundance might drive astrocytic proliferation and differentiation (Zeng et al., 2023; Bonneh-Barkay et al., 2011). Notably, increased levels of GFAP in the CSF have been linked to both AD (Jany et al., 2015) and multiple sclerosis (MS) (Kassubek et al., 2017).

Nevertheless, dual immunofluorescence assays revealed that not all plaque-associated GFAP-expressing astrocytes are YKL-40 positive (Moreno-Rodriguez et al., 2020). Further research on proteinopathies in the human brain has shown significantly elevated CSF levels of CHIT1, YKL-40, and GFAP, with a pronounced relationship between YKL-40 and GFAP concentrations and the presence of neurodegenerative markers (NFL, t-tau, p-tau, Aβ42, and Aβ40) (Abu-Rumeileh et al., 2019). The lack of a uniform correlation between YKL-40 and GFAP levels in AD patients suggests that these biomarkers may reflect discrete astrocytic subpopulations or their specific spatial configurations (Oeckl et al., 2019), indicating the need for further research to decode the complexities of these biomarker profiles.

## Mechanism of YKL-40 in AD pathogenesis

### The role of YKL-40 in neuroinflammation and neural apoptosis

Neuroinflammation, driven predominantly by inflammatory cytokines and neurotoxins, leads to neuronal degradation and death in specific areas of the central nervous system (Rama Rao & Kielian, 2015). This process can be triggered by various factors, including aging, trauma, stroke, bacterial toxins, and drugs, resulting in the exacerbation of neuritis through the production of inflammatory cytokines, complement, reactive oxygen species (ROS), and nitric oxide (NO). Central to the neuroinflammatory response are activated astrocytes and microglia, which mediate and regulate these processes (Singh, 2022). Introduction of proinflammatory agents *in vitro* or *in vivo* within AD mouse models has been shown to elicit deleterious effects on neuronal integrity, disrupting normal function and inciting apoptotic pathways. The upregulation of proinflammatory cytokines, most notably interleukin-1 beta (IL-1β) and interleukin-6 (IL-6), promotes the activation of astrocytes *via* Toll-like receptors (TLRs), and astrocytic activation is correlated with increased YKL-40 expression (Bonneh-Barkay et al., 2011; Li et al., 2021). YKL-40 exerts a distinct neurotoxic influence, where its presence has been shown to truncate neuronal processes and attenuate neural activity (Matute-Blanch et al., 2020). Additionally, YKL-40 is implicated in the suppression of the extracellular signal-regulated kinase 1/2 (ERK1/2) pathway, a critical cascade in AD pathophysiology associated with neuronal apoptosis. As depicted in the schematic, YKL-40 directly inhibits the binding of basic fibroblast growth factor (bFGF) to its receptor FGFR1, thereby blocking the bFGF signaling cascade. This inhibition, in turn, contributes to the suppression of the ERK1/2 pathway, which is pivotal for neuronal proliferation, differentiation, synaptic plasticity, and axonal growth. ERK, a pivotal component of the mitogen-activated protein kinase (MAPK) signaling pathway, predominantly resides within dendrites and axons, transmitting extracellular stimuli to the cellular nucleus. YKL-40’s interference with both the bFGF and ERK1/2 pathways underscores its potential role in the modulation of neuronal apoptosis and neurodegeneration (Xie et al., 2020; Liddelow et al., 2017). Compelling evidence from murine models deficient in YKL-40 delineates a complex role for this molecule in central nervous system injury and repair. Compared with their wild-type counterparts, YKL-40 knockout mice exhibit pronounced astrocyte proliferation and heightened immune cell infiltration following traumatic brain injury (Bonneh-Barkay et al., 2012b). Furthermore, the ablation of YKL-40 is correlated with a diminished amyloid plaque burden and the upregulation of the microglial lysosomal marker CD68 in plaques. These findings suggest that YKL-40 may impede the phagocytic ability of glial cells, thereby promoting the accumulation of Aβ (Lananna et al., 2020).

Investigations into the direct effects of YKL-40 on neurons have revealed its detrimental impact on neuronal viability and function. The treatment of primary murine cortical neurons with recombinant YKL-40 resulted in a discernible decrease in total axon length and extensive cell mortality within 48 h post-administration (Matute-Blanch et al., 2020). The underlying mechanism is complex, and the most widely accepted mechanism is as follows: the activation of GSK-3β promotes the binding of YKL-40 to CRTH2, and this binding further inhibits the expression of the β-catenin signalling pathway, thereby affecting the generation of hippocampal neurons (Jiang et al., 2023). Additionally, YKL-40 is implicated in the suppression of the extracellular signal-regulated kinase 1/2 (ERK1/2) pathway (Chen et al., 2021), a critical cascade in AD pathophysiology associated with neuronal apoptosis. ERK, a pivotal component of the mitogen-activated protein kinase (MAPK) signalling pathway, predominantly resides within dendrites and axons, transmitting extracellular stimuli to the cellular nucleus and is important for neuronal proliferation, differentiation, synaptic plasticity, and axonal growth. YKL-40 interferes with the ERK1/2 pathway, underscoring its potential role in the modulation of neuronal apoptosis (Areshkov et al., 2012).

**Figure 2:**
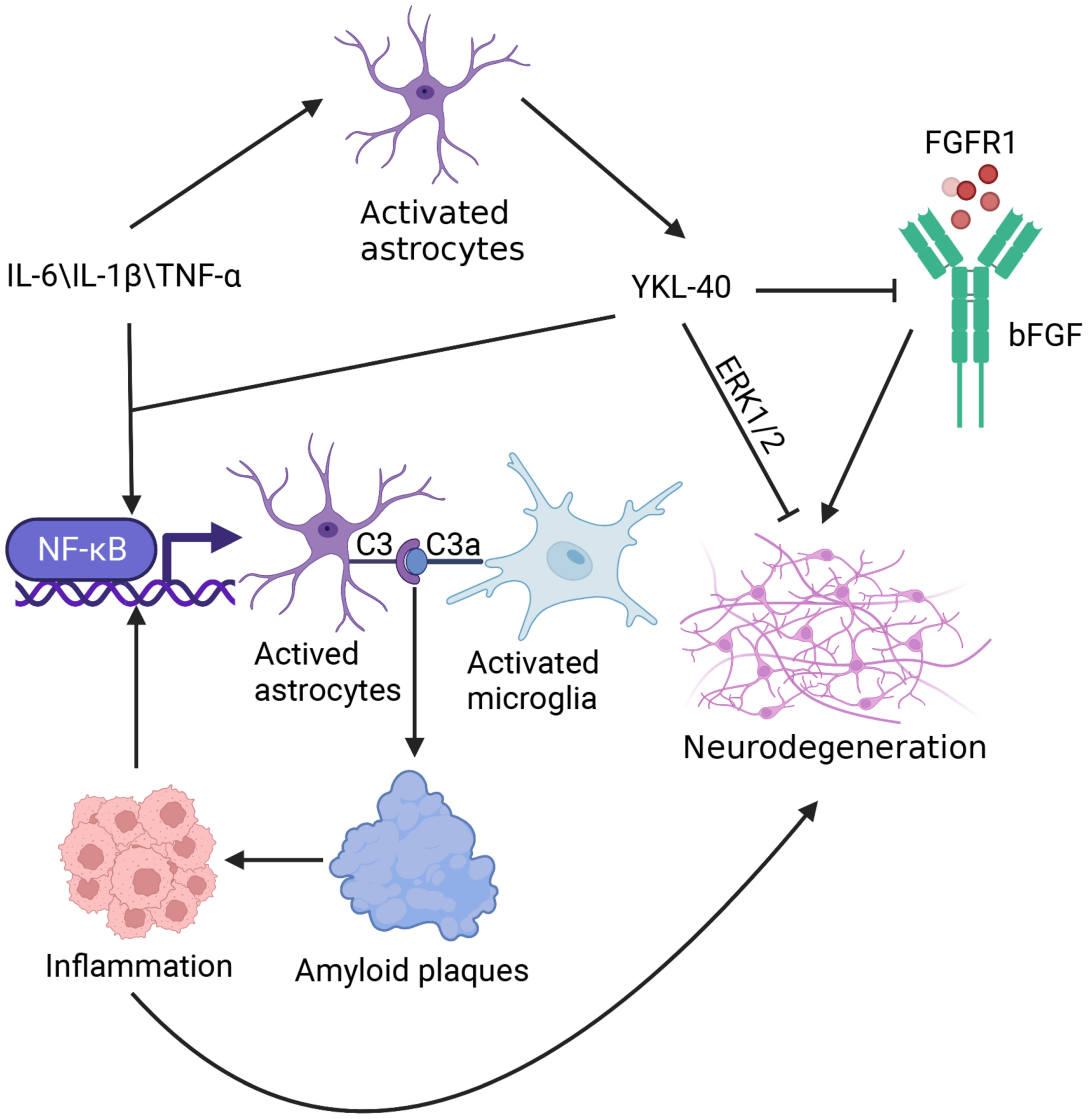
The roles of YKL -40 in neural inflammation and neurodegeneration in AD. Proinflammatory cytokines (IL-1β, IL-6, TNF-α) activate astrocytes *via* the NF-κ B signalling pathway, thereby upregulating the expression of YKL-40. YKL-40 exerts direct neurotoxic effects by inhibiting both the basic fibroblast growth factor (bFGF)-FGFR1 axis and the extracellular signal-regulated kinase 1/2 (ERK1/2) signalling cascade, which impairs neuronal survival and synaptic function. Concurrently, activated astrocytes and microglia synergistically amplify neuroinflammatory responses, promote amyloid-β (Aβ) plaque deposition, and induce oxidative stress. This forms a feedforward pathological cycle that accelerates neuronal damage, apoptosis, and the progression of AD.

In conclusion, the secretion of inflammatory factors such as IL-1β, IL-6, and TNF-α activates astrocytes, induces increased expression of YKL-40, leads to neuronal damage, promotes the accumulation of Aβ, and accelerates the process of AD. In addition, YKL-40 can inhibit the activation of the bFGF signalling pathway and the ERK1/2 pathway, which participate in neuronal apoptosis (Fig. 2).

### Regulation of YKL-40 expression in AD by the circadian clock

The circadian system is responsible for synchronizing 24-hour cycles in diverse physiological and behavioral aspects, such as sleep, activity patterns, and hormonal secretions (Mohawk, Green & Takahashi, 2012). Dysregulation of this system is often observed in patients with neurodegenerative disorders, including those in the preclinical stages of AD (Musiek et al., 2018). At the heart of the circadian mechanism lies a transcription−translation feedback loop driven primarily by the circadian locomotor output cycle kaput (CLOCK) gene and its partner BMAL1, which collectively modulates the timing of gene expression on a cell-specific basis. This biological timing system also plays a pivotal role in modulating immune responses across a spectrum of peripheral innate and adaptive immune cells (Curtis et al., 2014). Astrocytes and microglia, which are equipped with functional biological clocks, exhibit circadian variations in their inflammatory responses (Prolo, Takahashi & Herzog, 2005). A correlation study between sleep metrics and CSF biomarkers revealed a negative association between sleep efficiency and YKL-40 levels, a relationship persisting even after correcting for age, sex, and apolipoprotein E4 genotype (Targa et al., 2021).

Investigations using Bmal1 knockout (Bmal1 KO) mice revealed substantial downregulation of YKL-40 in the absence of Bmal1, whereas Period 1 (Per1) transcription was notably upregulated. These reciprocal expression dynamics in Bmal1- and Per-mutant mice suggest that YKL-40 could be under circadian control (Lananna et al., 2020). Furthermore, ablation of Bmal1 has been associated with the autonomous activation of astrocytes (Lananna et al., 2018). Given that YKL-40 expression is markedly suppressed in Bmal1KO astrocytes, this finding aligns with the observed decrease in Aβ plaque burden in YKL-40 KO mice, which parallels an augmented fibrillar plaque load consequent to a total loss of Bmal1 (Kress et al., 2018). These findings establish the role of the circadian clock in mediating the induction of YKL-40 in astrocytes. A global deficiency of Bmal1 extends its impacts to all brain cell types, perturbing peripheral clocks, disrupting sleep−wake cycles, and causing widespread rhythmical disturbances. Consequently, while YKL-40 expression does not exhibit a rhythmic pattern, it is unequivocally subject to the governance of the circadian clock system.

### The role of YKL-40 in the regulation of blood−brain barrier homeostasis

**Figure 3:**
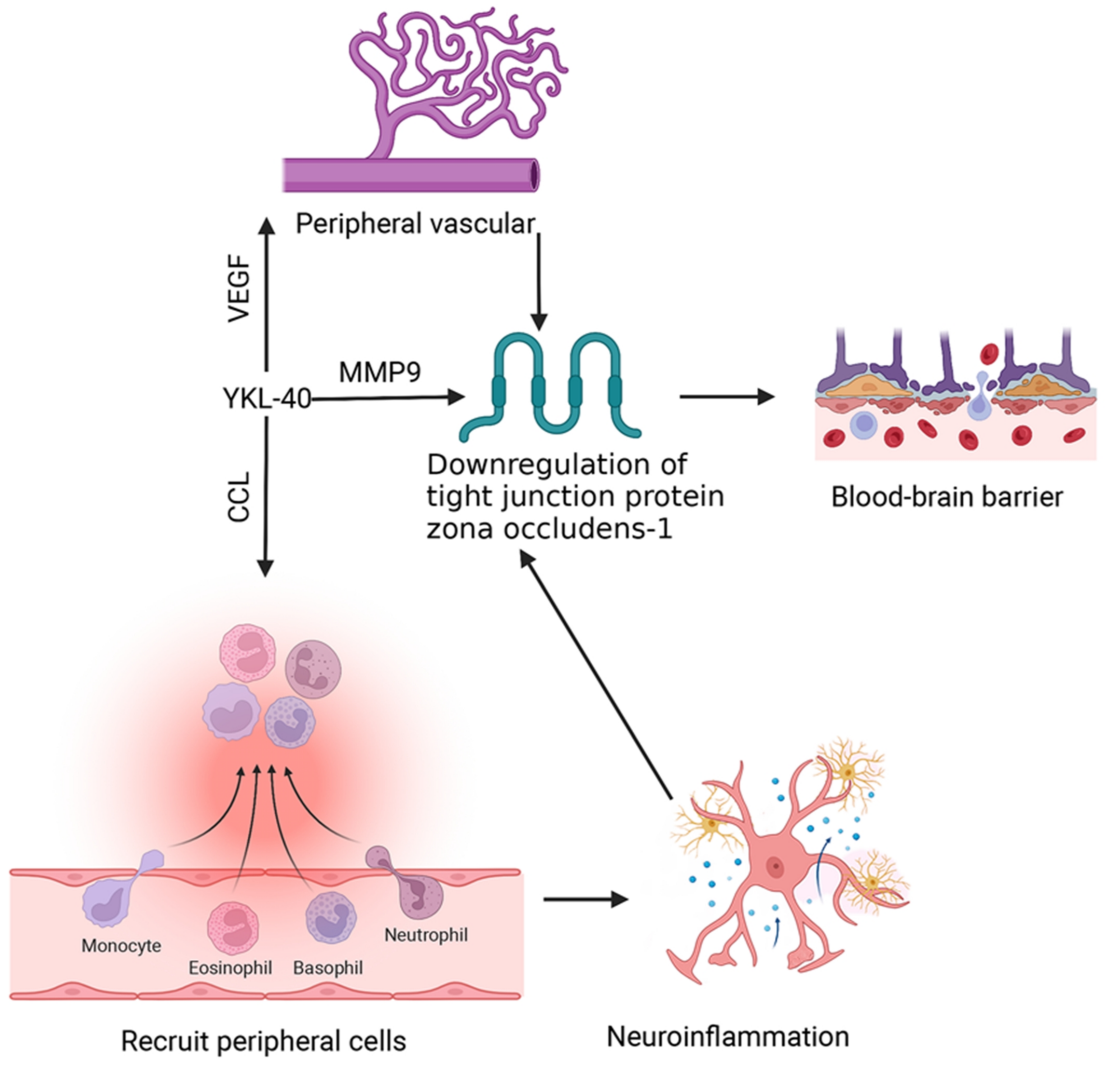
Possible roles of YKL-40 in the regulation of blood–brain barrier homeostasis. YKL-40 disrupts BBB integrity through multiple interconnected mechanisms: it upregulates vascular endothelial growth factor (VEGF) to promote peripheral angiogenesis and induces macrophages to synthesize matrix metalloproteinase 9 (MMP9) and chemokine CCL2. VEGF and MMP9 jointly downregulate the expression of the tight junction protein zonula occludens-1 (ZO-1), which increases BBB permeability. CCL2 recruits peripheral immune cells to infiltrate the central nervous system, amplifying neuroinflammation and further suppressing ZO-1 expression. Additionally, glial cell-derived inflammatory factors exacerbate tight junction damage, collectively impairing BBB homeostasis and facilitating AD pathogenesis.

The blood−brain barrier (BBB) constitutes a crucial interface between the cerebral vasculature and the neuronal milieu and is established by an intricate network of glial cells and the endothelial barrier of the choroid plexus (Hawkins & Davis, 2005). Its integrity is paramount for preserving neural homeostasis and the optimal functioning of the central nervous system. Emerging evidence from AD research points to early vascular alterations, notably preceding cognitive decline, with BBB dysfunction and metabolic disruptions being evident within the hippocampal region at the nascent stages of AD (Zlokovic, 2008; Sweeney, Sagare & Zlokovic, 2018). In patients with AD and MCI, the fidelity of BBB tight junctions is compromised, leading to diminished stability and heightened permeability, a condition intertwined with the levels of YKL-40 (Muszyński et al., 2017). This finding indicates that YKL-40 may be instrumental in the degradation of BBB integrity in AD. The albumin ratio, a hallmark of BBB disruption, is significantly associated with increased YKL-40 levels in the cerebrospinal fluid of AD patients, which is associated with BBB dysfunction (Muszyński et al., 2017). YKL-40 augments peripheral vascular genesis by upregulating VEGF, and VEGF, in turn, can attenuate the expression of the tight junction protein zonula occludens-1, potentially leading to BBB compromise (Li et al., 2024; Francescone et al., 2011; Suzuki, Nagai & Umemura, 2016). Moreover, YKL-40 has been shown to increase the synthesis of MMP9 and CCL2 in macrophages (Libreros et al., 2012). The MMP disrupts BBB integrity by disassembling basal lamina proteins and disintegrating tight junctions, whereas CCL2 mobilizes peripheral immune cells, which exacerbates inflammation and consequently inflicts damage upon the BBB (Fonseca et al., 2014). Concomitant with these peripheral immune elements, inflammation induced by glial cells can deleteriously influence the expression of tight junction proteins, culminating in BBB compromise (Fig. 3). While multiple studies have posited that YKL-40 contributes to BBB disintegration, the exact mechanisms of its involvement remain an area for exploratory research.

### The relationship between YKL-40 and glutamate transmission in neuronal physiology

Glutathione (GSH), an essential tripeptide comprising glutamic acid, cysteine, and glycine, is characterized by its γ-amide bonds and sulfhydryl groups and is ubiquitously distributed across nearly every cellular compartment within the human body. Research focusing on serum and plasma from patients diagnosed with MCI has shed light on the progressive oxidative deterioration of DNA, RNA, lipids, and proteins associated with these conditions (Williams et al., 2006; Pamplona et al., 2005). It is hypothesized that the oxidative stress observed in MCI and AD may stem from a depletion of antioxidant enzymes within the cerebral environment (Rinaldi et al., 2003). Verification of reduced GSH levels was achieved through *in vitro* studies (Ghosh et al., 2012), supported by *in vivo* experiments in animal models (Resende et al., 2008), and ultimately confirmed in postmortem analyses of AD brains (Venkateshappa et al., 2012). Complementary to these findings, diminished plasma GSH levels have also been reported in individuals with AD and MCI (Bermejo et al., 2008). Glutamate, an excitatory neurotransmitter, plays a critical role in regulating synaptic transmission and is essential for the functionality of the central nervous system, accounting for approximately half of all synaptic activity. Excessive glutamate has been associated with the activation of various extrasynaptic receptors in neurons and microglia, potentially leading to excitotoxicity and contributing to the neurodegenerative processes observed in AD.

Synaptic transmission mechanisms are facilitated by glutamate receptors, which include mainly NMDA receptors, AMPA receptors, and kainate receptors. The NMDA receptor, which serves as an ionic channel for the excitatory amino acid glutamic acid, is pivotal in several neural processes, both pathologically and physiologically, such as synaptic plasticity, learning, memory, ischemic neuronal death, neurodegeneration, epilepsy, and substance dependence. The AMPA receptor stands as a cornerstone for rapid excitatory synaptic transmission within the central nervous system, with its dynamic postsynaptic expression crucial for long-term potentiation and the initiation and maintenance of long-term depression, thereby playing a key role in learning and memory modulation. Kainate receptors (KARs), which constitute a significant class of ionotropic glutamate receptors, exert substantial regulatory effects on synaptic transmission and plasticity and are closely linked to fear memory encoding.

**Figure 4:**
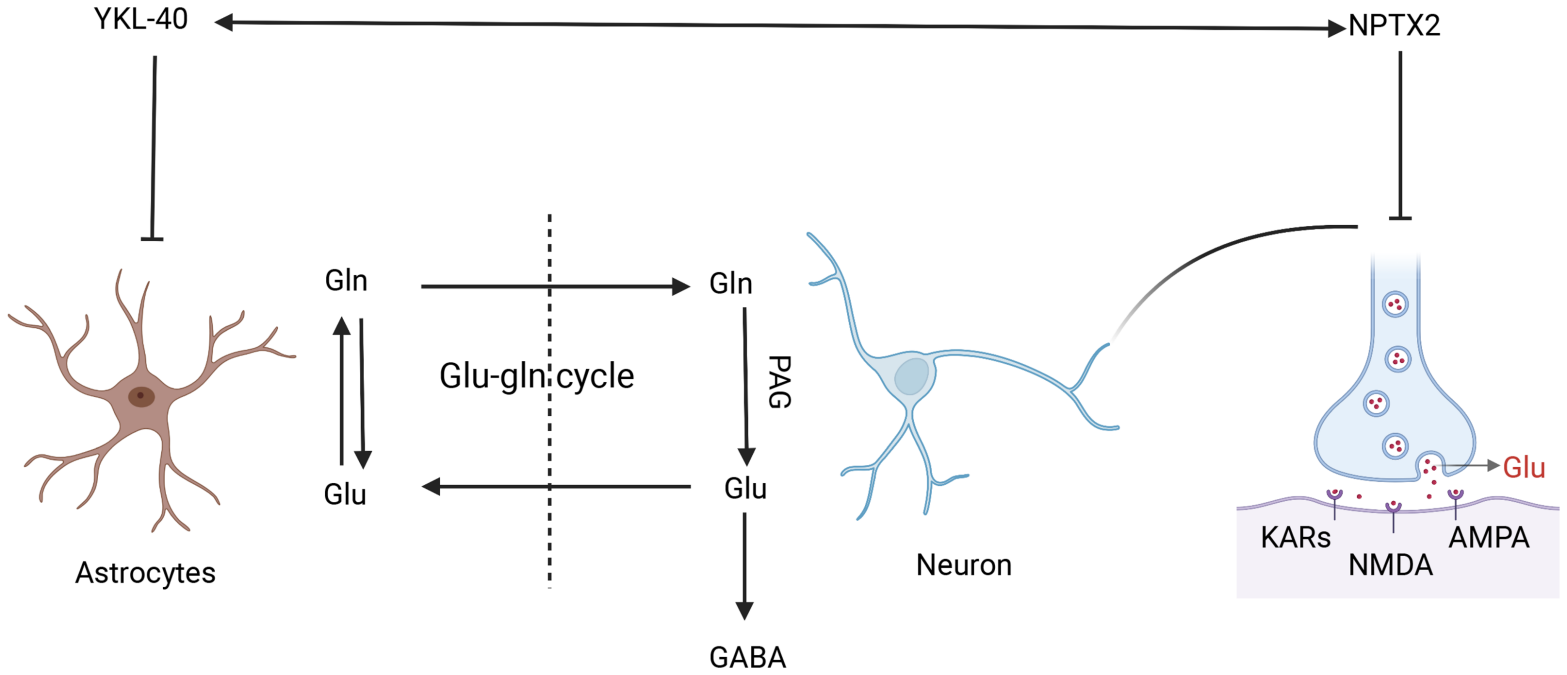
The modulation of glutamate transmission and synaptic function by YKL-40 in AD. YKL-40 is closely associated with astrocytic metabolic dysfunction, which disrupts the glutamate-glutamine (Glu-Gln) cycle. Astrocytes synthesize and secrete glutamine, which neurons convert to glutamate *via* phosphate-activated glutaminase (PAG). Glutamate released at synapses activates NMDA, AMPA, and kainate receptors to mediate excitatory synaptic transmission. YKL-40 interacts with pentraxin II (NPTX2) to destabilize glutamatergic synapses, and astrocytic metabolic disorders lead to glutamate accumulation, triggering neuronal excitotoxicity. Moreover, reduced glutathione (GSH) levels exacerbate oxidative stress, which further impairs synaptic plasticity and promotes neurodegeneration in AD.

In APP/PS1 murine models, a time-dependent increase in YKL-40 expression was observed, paralleling cognitive decline (Xiao et al., 2016). APP, through its interaction with β-secretase enzymes, may trigger neuronal hyperexcitability, potentially leading to epileptiform activity (Tambini, Yao & D’Adamio, 2019). Additionally, a significant correlation has been identified between YKL-40 and neural levels of pentraxin II (NPTX2), a member of the pentraxin family implicated in destabilizing hippocampal equilibrium by altering the formation and plasticity of glutamatergic synapses (Moreno-Rodriguez et al., 2020). Astrocytes are known for synthesizing and secreting glutamine, which neurons convert into glutamate *via* activated phosphate-activated glutaminase (PAG), an essential element of the glutamate/GABA-glutamine cycle. However, deletion of the PAG gene results in acute postnatal mortality in murine models (Masson et al., 2006). Investigations in McGill-r-thy1-app rat models of AD have revealed metabolic disturbances in astrocytes and disruptions in amino acid neurotransmitter balance, affecting glutamate and GABA neurotransmission and leading to neuronal death within the AD-afflicted brain (Nilsen, Witter & Sonnewald, 2014). These findings suggest that YKL-40 may be involved in the complex network of neuronal changes associated with AD, potentially through its interaction with glutamate-related processes (Fig. 4).

Elevated YKL-40 levels are indicative of astrocytic metabolic dysregulation. Research on hippocampal and cerebral cortical metabolites in rodents with AD has demonstrated that trocytic metabolic dysfunction leads to impairments in glutamate–glutamine metabolism, oxidative metabolism, the tricarboxylic acid cycle, mitochondrial function, and glucose utilization. These disruptions subsequently impair GABA synthesis in cerebral neurons and precipitate neuronal death (Andersen, Schousboe & Verkhratsky, 2022; McCann & Maguire-Zeiss, 2021; Mi et al., 2023). Despite the established associations between YKL-40 and these metabolic alterations, comprehensive research on the direct metabolic interplay between YKL-40 and glutamic acid is currently lacking. The precise impacts of YKL-40 on neurons in this context remain to be fully elucidated. Therefore, further investigations are essential to gain a more in-depth understanding of the underlying mechanisms and to clarify the role of YKL-40 in the complex relationship between glutamate metabolism and neuronal death in AD.

## Targeting YKL-40 for the treatment of AD

### YKL-40 and its receptors: unravelling their significance in AD

Mammalian YKL-40 is a secreted glycoprotein that belongs to the glycoside hydrolase family 18 in the CAZy database on the basis of its significant homology with this enzyme family (Houston et al., 2003; Fusetti et al., 2003; Lombard et al., 2014). Despite the fact that YKL-40 manifests a certain level of structural similarity to the glycoside hydrolase family 18, the catalytically active glutamate and aspartate motifs, which are quintessentially emblematic of the glycoside hydrolase family 18, are located within YKL-40. This substitution, consequently, abrogates the catalytic activity of YKL-40. As a corollary of this forfeiture of catalytic function, YKL-40 is thereby transmuted into a lectin, which is designated a noncatalytic sugar-binding protein. Structural evidence that YKL-40 encompasses at least two functional binding domains is currently available. The principal binding cleft of YKL-40 contains nine binding subsites, on which aromatic residues are arranged in a manner compatible with carbohydrate binding. Additionally, a putative heparin-binding site located within a surface loop has been proposed. However, *in vitro* binding affinity studies have not been able to definitively confirm the presence and functionality of this heparin-binding site (Houston et al., 2003).

To date, a total of six receptors for YKL-40 have been identified, specifically interleukin-13 receptor subunit α-2 (IL-13Rα2), transmembrane protein 219 (TMEM219), galectin-3 (Gal-3), CD44, chemoattractant receptor-homologous molecule expressed on Th2 cells (CRTH2), and receptor for advanced glycation end products (RAGE) (Lee et al., 2016). YKL-40 has come to the forefront as a prospective mediator of AD, and its engagement with particular receptors has been implicated in diverse pathological cascades.

#### YKL-40 and IL-13Rα2

IL-13Rα2, a receptor subunit involved in the interleukin-13 (IL-13) signalling pathway, plays a significant role in various immune responses and inflammatory processes. In AD, IL-13Rα2 has been implicated in the activation of the mitogen-activated protein kinase/extracellular signal-regulated kinase (MAPK/ERK) pathway, the protein kinase B (AKT/PKB) pathway, and the Wnt/β-catenin signalling pathway. The interaction between YKL-40 and IL-13Rα2 is suggested to be associated with the progression of AD, potentially influencing tau hyperphosphorylation and the formation of NFTs, which are key pathological features of the disease (Lee et al., 2016).

The aberrant activation of the MAPK/ERK pathway, which results from the interaction between YKL-40 and IL-13Rα2, is implicated in tau hyperphosphorylation. Hyperphosphorylated tau disengages from microtubules and aggregates to form NFTs. These NFTs disrupt neuronal microtubule networks, impede axonal transport processes, and culminate in neuronal dysfunction and cell death. As an illustrative example, NFTs are prevalent in the hippocampal and cortical regions of AD patients and are of paramount importance for learning and memory functions. The progressive accumulation of NFTs in these regions leads to a successive decline in cognitive ability and impairment of cognitive function (Kirouac et al., 2017; Griffin et al., 2005).

In addition, research findings have shown that the AKT/PKB pathway activated by YKL-40 may disrupt neuronal energy metabolism in AD by interfering with glucose uptake and metabolism, affecting mitochondrial function and altering the activity of metabolism-related enzymes. Neurons require substantial energy for maintaining physiological functions, and abnormal AKT/PKB signalling can alter energy substrate uptake and utilization, rendering neurons vulnerable to Aβ toxicity. Aβ accumulation, a hallmark of AD, further impairs neuronal function and clearance mechanisms, establishing a vicious cycle of neurodegeneration and cognitive decline (Griffin et al., 2005)

**Figure 5:**
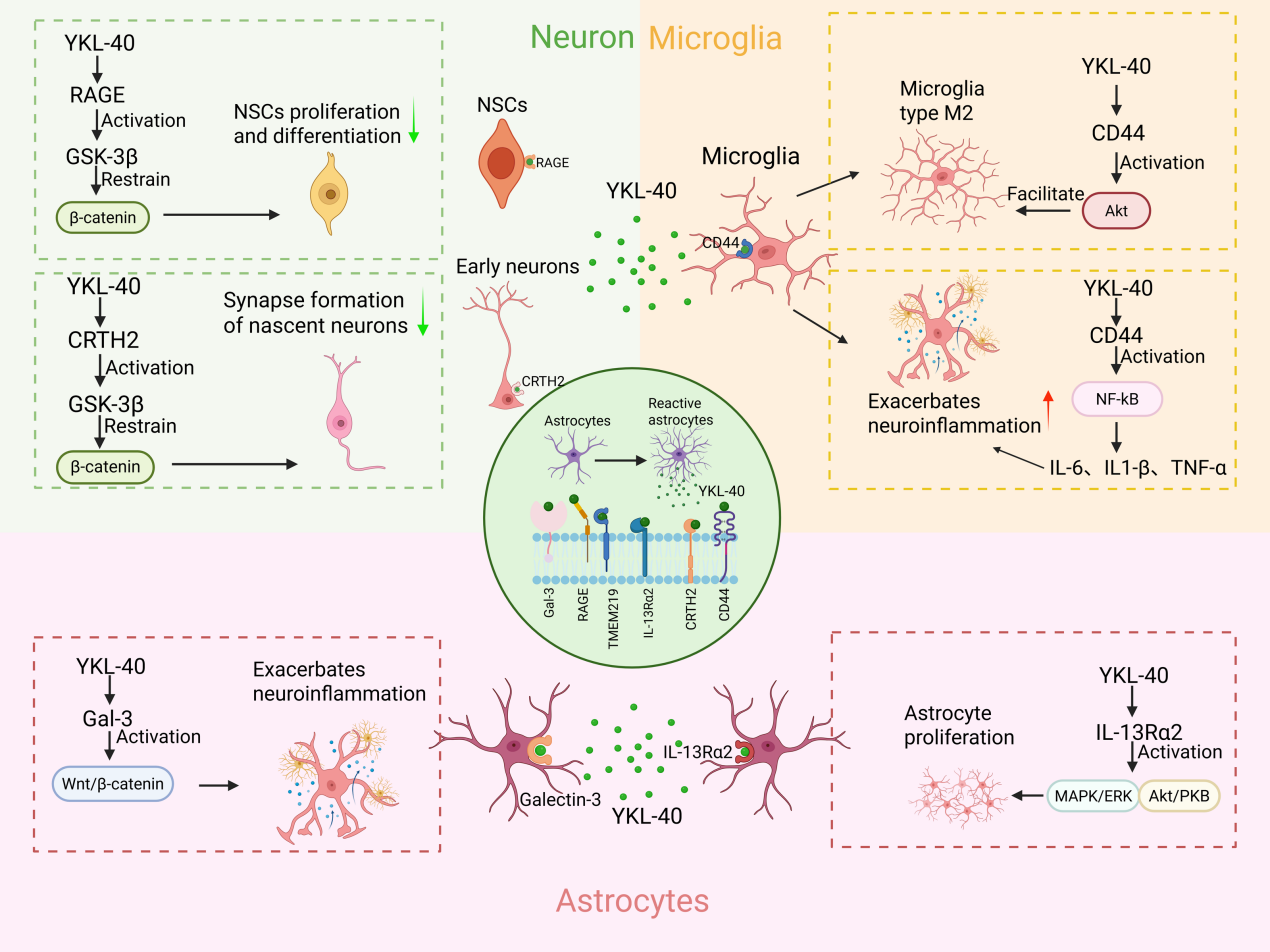
YKL-40 interactions and pathways in Alzheimer’s Disease Pathogenesis. YKL-40 binds to six core receptors (IL-13Rα2, TMEM219, Gal-3, CD44, CRTH2, RAGE) to activate diverse downstream signalling pathways, including MAPK/ERK, AKT/PKB, Wnt/β-catenin, and NF-κ B. Binding to IL-13Rα2 and TMEM219 drives tau hyperphosphorylation and neurofibrillary tangle (NFT) formation *via* the MAPK/ERK pathway, and disrupts neuronal energy metabolism through AKT/PKB signalling. Gal-3 and CD44-mediated Wnt/β-catenin pathway dysregulation impairs synaptic plasticity and neural stem cell repair capacity. RAGE binding uniquely activates NF-κ B, which induces glial cell activation and proinflammatory cytokine release, forming a positive feedback loop that amplifies neuroinflammation. Collectively, these cascades promote Aβ deposition, synaptic loss, and neuronal apoptosis, accelerating AD progression.

The Wnt/β-catenin signalling pathway is essential for neuronal development and synaptic plasticity (Shen et al., 2022; Lv et al., 2023). In AD, dysregulation of this pathway following (He et al., 2013) binding leads to synaptic loss and cognitive impairment. Abnormal activation can disrupt synaptic formation and stability, impairing neuronal communication and cognitive function (Song et al., 2024). Additionally, it may affect neural stem cell proliferation and differentiation, reduce the self-repair capacity of the brain and exacerbate AD progression (He et al., 2013; Bahlakeh et al., 2022) (Fig. 5).

#### YKL-40 and TMEM219

TMEM219 is a membrane protein that has been implicated in the regulation of immune responses and cellular signalling pathways. It is known to interact with the IL-13Rα2/YKL-40 complex, enhancing the binding affinity of IL-13 and playing a pivotal role in the activation of the MAPK/ERK and AKT/PKB signalling cascades. However, TMEM219 does not influence the Wnt/β-catenin pathway (Lee et al., 2016). In AD, TMEM219-mediated regulation of the MAPK/ERK and AKT/PKB pathways influences neuronal apoptosis and oxidative stress. Dysfunction of TMEM219 can lead to imbalanced activation of these pathways, increasing neuronal susceptibility to oxidative stress-induced apoptosis. Oxidative stress elevates reactive oxygen species (ROS) levels, damages cellular components and disrupts neuronal function. Aberrant MAPK/ERK activation can further exacerbate oxidative stress, creating a self-perpetuating cycle of neuronal damage (Rezatabar et al., 2019). TMEM219 is also involved in regulating melanoma metastasis and TGF-β1 production. TGF-β1 has diverse functions in the nervous system, and its dysregulation due to TMEM219 abnormalities may disrupt the inflammatory microenvironment of the brain and impact neuronal survival (Marino et al., 2022). In summary, the interaction of TMEM219 with YKL-40 and its role in signalling pathways associated with neuronal survival and the oxidative stress response are key factors in AD pathogenesis (Fig. 5).

#### YKL-40 and Gal-3

Gal-3, also known as galectin-3, is a multifunctional protein involved in various physiological and pathological processes, including cell activation, proliferation, and migration. It is a soluble β-galactoside-binding protein that can promote apoptosis when located extracellularly and influence macrophage differentiation and fibrotic repair when inside the cell. Gal-3 has been implicated in the regulation of synaptic function and cognition in AD. YKL-40 binding to Gal-3 activates the Wnt/β-catenin signalling pathway (Zhou et al., 2018). Gal-3 performs different functions depending on its intracellular or extracellular localization, promoting apoptosis extracellularly and influencing macrophage differentiation and fibrotic repair intracellularly. Intracellular Gal-3 affects macrophage differentiation and fibrotic repair (Coulis et al., 2023). Macrophages play a crucial role in Aβ clearance, and abnormal Gal-3-mediated macrophage differentiation can impair this function, leading to Aβ deposition and plaque formation (Sun et al., 2024; Krasemann et al., 2017). Fibrotic repair processes in the brain may also be altered, potentially affecting neuronal survival and function. Excessive fibrosis can form physical barriers, impeding neuronal signalling and nutrient exchange. As mentioned, the Wnt/β-catenin pathway is vital for synaptic function and cognition, and YKL-40/Gal-3-induced activation of this pathway may disrupt its normal regulation in AD, leading to synaptic plasticity abnormalities, reduced synaptic connections and transmission efficiency, and further cognitive impairment (Fig. 5). Further research into the mechanisms by which Gal-3 and YKL-40 interact and their impact on AD pathology is essential for developing effective treatments.

#### YKL-40 and CD44

CD44, a transmembrane glycoprotein that plays a pivotal role in cell proliferation, viability, and differentiation, has been shown to interact with IL-13Rα2, initiating the MAPK/ERK, AKT/PKB, and Wnt/β-catenin signalling pathways (Geng et al., 2018). This interaction is crucial for modulating neuroinflammation in AD. The activation of microglia and astrocytes, which are central to the neuroinflammatory response, can be significantly influenced by CD44-mediated signalling. In AD, the aberrant activation of these glial cells due to abnormal CD44 signalling can lead to the overproduction of inflammatory cytokines such as TNF-α, IL-1β, and IL-6 in neurons (Liu et al., 2014). These cytokines can directly or indirectly contribute to neuronal damage, exacerbating the neuroinflammatory environment and potentially driving disease progression. Moreover, CD44 signalling has been implicated in the regulation of neuronal function and survival. Disruption of CD44 signalling can result in increased vulnerability of neurons to oxidative stress, which is characterized by increased levels of ROS that can damage cellular components and impair neuronal function. While the direct role of CD44 in AD is still under investigation, its influence on cell−cell interactions and inflammation modulation suggests a potential indirect contribution to AD pathology.

#### YKL-40 and CRTH2

CRTH2, also known as the prostaglandin D2 (PGD2) receptor, is a G protein-coupled receptor that is involved in the immune response, particularly in type 2 inflammation. The activation of CRTH2 can lead to proinflammatory actions, such as the migration and degranulation of immune cells. In AD, CRTH2 may play a role in modulating the immune response and inflammation within the central nervous system. YKL-40, upon binding to CRTH2, has been shown to increase collagen accumulation and fibrotic responses, which are key contributors to the pathogenesis of AD (Fig. 5). Although the precise mechanisms by which CRTH2 operates within the YKL-40 signalling cascade have not been fully elucidated, its association with Hermansky-Pudlak mutant cells and the noted reduction in IL-13Rα2 membrane expression suggest a complex interplay (Zhou et al., 2015). The accumulation of collagen and the occurrence of fibrosis can profoundly impact the composition and architecture of the extracellular matrix. The extracellular matrix plays a pivotal role in preserving neuronal morphology, buttressing synaptic functionality, and modulating intercellular communication. Aberrant collagen deposition and fibrosis can lead to stiffening of the extracellular matrix, thereby impeding the growth and arborization of neuronal axons and suppressing synaptic plasticity. Consequently, these pathological changes disrupt the transmission and integration of neural signals (Dan & Zhang, 2023; Logsdon et al., 2021). Fibrotic tissue can also form physical barriers, impede nutrient and metabolite diffusion and affect neuronal metabolism and function.

Moreover, CRTH2 may influence the behavior of immune cells and the progression of neuroinflammation. The interaction between CRTH2 and YKL-40 could modulate the migration and distribution of immune cells within the brain, affecting their response to pathological stimuli. This interaction may also disrupt the balance between immune cells and neurons, impacting the release and clearance of inflammatory factors and exacerbating the neuroinflammatory environment, which could lead to severe neuronal damage and contribute to disease progression (Jiang et al., 2023).

#### YKL-40 and RAGE

RAGE, or receptor for advanced glycation end products, is a multiligand receptor that plays a significant role in the pathogenesis of AD. RAGE is known to bind to a variety of ligands, including advanced glycation end products (AGEs), which are formed when proteins or lipids undergo nonenzymatic glycation reactions. RAGE has been implicated in the progression of this disease by promoting neuroinflammation and neuronal damage.

YKL-40 binding to RAGE activates several signalling pathways, including the MAPK/ERK, Wnt/β-catenin, NF-κB, and STAT3 pathways (Song et al., 2024; Mizoguchi & Wang, 2025). This interaction is particularly noteworthy, as RAGE is the only known receptor capable of activating NF-κB, a key transcription factor in neuroinflammation. In AD, YKL-40-RAGE-induced NF-κB activation leads to the production of numerous proinflammatory cytokines (Fig. 5). Under NF-κB activation, microglia and astrocytes release TNF-α, IL-1β, IL-6, and inducible nitric oxide synthase (iNOS), triggering an inflammatory cascade (Chae et al., 2018). This inflammatory environment directly damages neurons, disrupting membrane integrity, mitochondrial function, and neurotransmitter metabolism. Chronic neuroinflammation also promotes Aβ aggregation and tau phosphorylation, exacerbating AD pathology (Ali et al., 2023).

The STAT3 pathway is involved in immune regulation and cell survival. In AD, YKL-40-activated STAT3 may affect immune cell function and neuronal survival. STAT3 activation can modulate the immune phenotype of microglia and astrocytes, and abnormal signalling can lead to immune cell dysfunction and an inability to provide neuroprotection. STAT3 also regulates neuronal survival signals, and abnormal activation can disrupt apoptotic programs, leading to either abnormal neuronal survival or death. Both scenarios can disrupt neuronal homeostasis and exacerbate AD symptoms (Jin et al., 2022; Xu et al., 2024; Yun et al., 2021).

The positive feedback loop between YKL-40 and NF-κB amplification through RAGE binding exacerbates AD pathology. Increasing YKL-40 levels continuously activate signalling pathways, intensifying neuroinflammation, neuronal damage, and other pathological processes. Elevated YKL-40 further activates NF-κB, leading to increased inflammatory factor production, a more severe inflammatory reaction, and subsequently increased YKL-40 expression, establishing a vicious cycle that accelerates AD progression and impairs brain function (Chae et al., 2018; Lin et al., 2024).

Understanding the role of RAGE in YKL-40 signalling and its impact on AD progression is crucial for developing effective therapeutic strategies. Targeting the RAGE-YKL-40 interaction and its associated signalling cascades may provide a novel approach to mitigate neuroinflammation and promote neuronal integrity in AD. Further research into the mechanisms by which RAGE influences AD pathogenesis could reveal new avenues for therapeutic intervention.

### YKL-40-targeted agents with potential for AD therapy

Significant progress has been made in the development of therapeutic strategies targeting YKL-40 in preclinical studies. Multiple YKL-40 binders or inhibitors, especially those in the category of natural compounds such as chitin, ebractenoid F-like compounds, and G721-0282, have been proven to be able to effectively interfere with the biological functions of YKL-40. Moreover, investigations have demonstrated that the chitin-binding domain of compound K284-6111 with YKL-40 is capable of effectively suppressing the binding between YKL-40 and its receptor IL-13Rα2. Additionally, interfering RNAs can also be directed towards YKL-40 (Fan et al., 2024). Undoubtedly, these studies provide potential research avenues for the development of therapeutic agents for AD.

#### Chitosan as natural substrates of YKL-40

Chito-oligosaccharides, which are natural substrates of YKL-40, play a significant role in the interaction with this glycoprotein. YKL-40, a member of the chitinase protein family, specifically binds to both short and long chitooligosaccharides, suggesting preferential site selection on the basis of its affinity (Houston et al., 2003; Fusetti et al., 2003; Mizoguchi, 2006). In line with the characteristics of the glycoside hydrolase family 18, YKL-40 specifically binds to both short and long chitooligosaccharides, suggesting that it undergoes affinity-based preferential site selection. The binding of chitosan to YKL-40 is hypothesized to induce conformational changes in the protein, which may be crucial for its biological functions. This interaction could be pivotal in the development of novel therapeutic strategies targeting YKL-40 in AD. Understanding the specific interaction between YKL-40 and its natural ligand, chito-oligosaccharides, may provide valuable insights into the development of novel therapeutic strategies that target YKL-40 in AD. By modulating the binding of YKL-40 to chitooligosaccharides or interfering with the conformational changes induced by this binding, disrupting the downstream signalling pathways associated with YKL-40 and thereby attenuating AD pathology may be possible. A comprehensive elucidation of these signalling pathways has the potential to reveal novel avenues for early intervention and preventative strategies not only for AD patients and those at risk but also potentially for a broader spectrum of neurodegenerative diseases.

#### Inhibitors of YKL-40

Inhibitors of YKL-40, such as G721-0282 and K284-6111, have emerged as promising therapeutic agents for AD. G721-0282 is usually used to study small molecule compounds related to immune system responses, which are associated with the activation of certain immune cells, immune responses, or certain diseases (such as cancer and inflammatory diseases). In mice subjected to chronic unpredictable mild stress (CUMS) treatment, G721-0282 acts as an inhibitor of YKL-40 and has anxiolytic-like effects. Studies have shown that the administration of G721-0282 can relieve CUMS-induced anxiety, and its anxiolytic-like effect is related to the decreased expression of inflammatory proteins and cytokines induced by CUMS in the hippocampus. Both *in vivo* and *in vitro* experiments have shown that G721-0282 can inhibit the elevated levels of YKL-40 and IGFBP3 induced by CUMS. However, YKL-40 deficiency eliminated the anti-inflammatory effect of G721-0282 in microglial BV-2 cells. These findings indicate that G721-0282 may reduce CUMS-induced anxiors by inhibiting YKL-40-mediated neuroinflammation and regulating IGFBP3 (Ham et al., 2022).

K284-6111 is an orally active inhibitor of YKL-40 that significantly reduces the expression of inflammatory proteins and inhibits β-secretase and NF-κB, which are key mediators of neuroinflammation in AD. By reducing the phosphorylation of IκB, K284-6111 diminishes the nuclear translocation of NF-κB subunits, thereby inhibiting the expression of NF-κB-related neuroinflammatory genes. This results in decreased protein levels of COX-2, iNOS, GFAP, and Iba-1, as well as reduced mRNA levels of proinflammatory cytokines such as TNF-α, IL-1β, and IL-6, and the inflammatory factor NO (Ham et al., 2020). K284-6111 specifically inhibits the translocation of the p65 subunit in BV-2 microglia and astrocytes, which are pivotal in the neuroinflammatory response in AD. In addition to its effects on neuroinflammation, K284-6111 has multiple benefits on AD-related pathological processes. It reduces the expression of BACE1 and APP in LPS-treated BV-2 cells and cultured astrocytes, potentially decreasing Aβ production. In AD model mice, K284-6111 attenuates increased β-secretase activity and amyloid plaque formation, leading to significant memory-restorative effects. Studies on Tg2576 AD transgenic mice treated with K284-6111 revealed a decrease in p-ERK levels, suggesting the involvement of the ERK signalling pathway in the inhibitory effect of K284-6111 on neuroinflammation. K284-6111 also decreases APP and BACE1 levels, β-secretase activity, and Aβ plaque accumulation in Tg2576 transgenic mice, alleviates memory impairment, and improves cognitive dysfunction, further highlighting its therapeutic potential for AD (Lee et al., 2021) (Table 1).

In summary, inhibitors of YKL-40, such as G721-0282 and K284-6111, offer a novel approach to modulating neuroinflammation and amyloidogenesis in AD, with potential therapeutic benefits that extend to alleviating cognitive dysfunction and improving memory. These findings underscore the importance of YKL-40 as a therapeutic target in the treatment of AD.

#### Small interfering RNA targeting YKL-40

Some studies have shown that small interfering RNAs (siRNAs) targeting YKL-40 (siYKL-40) can inhibit the proliferation, migration, and invasion of human endometrial cancer (EC) HEC-1A cells and increase their antiapoptotic ability. In AD, YKL-40 interfering RNA may inhibit angiogenesis by regulating the vascular endothelial growth factor (VEGF)/VEGFR2 and ERK1/2 signalling pathways. Specifically, siYKL-40 can reduce the level of VEGFA and tube formation in endothelial cells and simultaneously decrease the expression levels of VEGF, phosphorylated vascular endothelial growth factor receptor 2 (pVEGFR2), and phosphorylated extracellular signal-regulated kinase 1 and 2 (p-ERK1/2) (Chen et al., 2021). In addition, an increasing number of studies support the neuroinflammation hypothesis of AD. The brains of AD patients exhibit chronic inflammation, which may be triggered by insoluble amyloid β deposits and neurofibrillary tangles (NFTs) and is associated with the activation of a cascade leading to neuronal death. Research has shown that the levels of YKL-40 in cerebrospinal fluid are related to the diagnosis of AD. YKL-40, a 40 kDa glycoprotein secreted by astrocytes, may play a role in the development of AD by regulating neuroinflammatory responses through its interference RNA (Mavroudis et al., 2021).

**Table 1:** Summary of inhibitors for YKL-40.

**Inhibitor name**	**Source/Type**	**Mechanism of action**	**Test models**	**Key preclinical findings**	**References**
**G721-0282**	Synthetic small molecule	Binds to YKL-40’s chitin-binding domain, inhibits YKL-40 dimerization	***In Vivo***: Chronic Unpredictable Mild Stress (CUMS) mouse model ***In Vitro***: Microglial BV-2 cell line	↓Hippocampal YKL-40 expression by 38%; ↓ Proinflammatory cytokines (IL-1β/IL-6) by 25–30%	Jin et al. (2022)
**K284-6111**	Orally active synthetic	Blocks YKL-40-IL-13Rα2 interaction; inhibits NF-κB nuclear translocation	***In Vivo***: Tg2576 AD transgenic mouse model ***In Vitro***: BV-2 microglial cells, astrocytes	↓Aβ plaque burden by 45%; ↓BACE1activity by 32%; Rescues spatial memory impairment	Xu et al. (2024) and Yun et al. (2021)

**Notes.**

Abbreviations YKL-40 Chitinase-3-like protein 1 AD Alzheimer’s disease CUMS Chronic unpredictable mild stress LPS Lipopolysaccharide IL-1β Interleukin-1β IL-6 Interleukin-6 NF-κB Nuclear factor kappa-light-chain-enhancer of activated B cells BACE1 β-site amyloid precursor protein cleaving enzyme 1

In a nude mouse endometrial carcinoma xenograft model, silencing the YKL-40 gene inhibited tumor growth and reduced the expression of CD68. Similarly, in AD animal models, YKL-40 interference RNA may have an impact on disease progression. For example, in AD mouse models, studying the effects of YKL-40 interference RNA on amyloid plaque formation, tau protein tangles, and neuronal apoptosis could provide new therapeutic targets for AD. In addition, in a mouse asthma model, adenovirus-mediated YKL-40 short hairpin RNA (shRNA) can alleviate eosinophilic airway inflammation, airway hyperresponsiveness, and airway mucus secretion and reduce YKL-40 levels in bronchoalveolar lavage fluid (BALF) and serum. Moreover, the levels of IL-5 and IL-13 in the lung tissues of asthmatic mice, as well as the levels of eosinophil-associated chemokines (eotaxin) and granulocyte−macrophage colony−stimulating factor (GM−CSF) in the BALF and serum, are significantly decreased (Wang et al., 2020). These findings suggest that in AD animal models, YKL-40 interference RNA may influence disease progression by regulating immune responses. Further research on the effects of YKL-40 interference RNA on YKL-40 levels in the cerebrospinal fluid or blood of AD patients, as well as its relationship with disease diagnosis and prognosis, could provide new insights for the clinical diagnosis and treatment of AD.

## Conclusion and Prospects

In the incipient stages of AD, the presence of YKL-40 in CSF is notable and escalates over time, concurrent with a gradual decrease in cognitive function. The concentration of the YKL-40 protein in the CSF of individuals with AD increased, exhibiting a positive correlation with the levels of the hallmark AD pathogenic proteins Aβ and tau—a key clinical observation that links YKL-40 to core AD pathology and cognitive decline (Chen, Shi & Li, 2025). Predominantly expressed by activated astrocytes within the brain, YKL-40 may expedite alterations in neuronal transmission through the modulation of neuroinflammatory processes, blood−brain barrier integrity, and glutamate trafficking, collectively contributing to the pathogenesis of AD.

The therapeutic landscape for neurodegenerative ailments, especially AD, presents formidable challenges, among which YKL-40 has emerged as a highly clinically relevant target due to its direct involvement in AD’s pathological cascade and correlation with cognitive impairment (Sortino, Amato & Musumeci, 2024). *In vitro* studies have shown that a series of YKL-40 binding agents or inhibitors, especially natural compounds, have potential for the treatment of AD. Among them, chitin, a naturally abundant polysaccharide, specifically interacts with YKL-40 *in vitro via* its unique chemical and physical properties, blocking YKL-40’s ability to promote neuroinflammation and Aβ aggregation—two central drivers of AD progression. The ebractenoid F-like compound precisely targets YKL-40 *in vitro* through its specific active groups and molecular conformations, inhibiting YKL-40-mediated signal transduction pathways that exacerbate tau hyperphosphorylation and synaptic dysfunction, thus directly mitigating key pathological processes linked to cognitive decline. In addition, the novel compound G721-0282 has been confirmed in detailed *in vitro* experiments to act on YKL-40 with high specificity; by blocking its key active sites or interfering with its binding to upstream/downstream pro-inflammatory and pro-pathological molecules, it abrogates YKL-40’s capacity to disrupt blood−brain barrier integrity and promote glutamate excitotoxicity—critical mechanisms underlying neuronal loss and cognitive impairment.

Notably, *in vitro* studies demonstrate that YKL-40-targeting small interfering RNA inhibits angiogenesis *via* modulation of the VEGF/VEGFR2 and ERK1/2 pathways, suppressing neurovascular dysfunction that contributes to Aβ clearance impairment and cognitive decline in AD. As a 40-kDa glycoprotein secreted by astrocytes, YKL-40 acts as a key driver of AD pathogenesis by regulating neuroinflammatory responses *in vivo*; thus, silencing YKL-40 specifically targets this microglia-astrocyte crosstalk axis, breaking the cycle of neuroinflammation, Aβ/tau pathology, and synaptic loss to halt or reverse cognitive deterioration (Chen et al., 2021).

Crucially, YKL-40-targeted therapies exhibit specificity in modifying AD progression by directly addressing root pathological causes rather than merely alleviating symptoms: by suppressing YKL-40 activity, these interventions specifically reduce excessive neuroinflammation that fuels Aβ aggregation and tau hyperphosphorylation, mitigate Aβ/tau pathology by disrupting YKL-40’s pro-aggregatory and pro-toxic functions, preserve synaptic integrity and transmission by blocking YKL-40-mediated glutamate excitotoxicity and blood−brain barrier disruption, and ultimately improve cognitive outcomes—including memory, attention, and executive function—that are most impactful for AD patients’quality of life (Gispert et al., 2016; Hellwig et al., 2015). Nevertheless, the role of YKL-40 in neuroinflammation remains debated: some *in vitro* investigations report anti-inflammatory effects of YKL-40, while YKL-40 knockout animal models show moderately increased proinflammatory cytokine expression and exacerbated neuroinflammation (Lananna et al., 2020). These discrepancies highlight the context-dependent complexity of YKL-40’s function, underscoring the need for further *in vitro* and *in vivo* combined experiments to refine therapeutic strategies—particularly to ensure YKL-40 inhibition specifically targets its pathogenic isoforms or activities without disrupting potential beneficial roles.

The exploration of YKL-40 signalling and its role in AD pathological progression will drive more exhaustive research. A comprehensive elucidation of these pathways has the potential to reveal novel, clinically actionable avenues for early intervention (in at-risk individuals) and disease-modifying treatment (in symptomatic patients), as YKL-40-targeted therapies offer the unique advantage of specifically interrupting the AD pathological cascade to preserve cognitive function, slow disease progression, and potentially reverse early-stage deficits—addressing the unmet clinical need for therapies that meaningfully improve patient outcomes.

## Additional Information and Declarations

### Data Availability

The following information was supplied regarding data availability: This is a Literature Review.
